# Supplementary material for: Flecainide Specifically Targets the Monovalent Countercurrent Through the Cardiac Ryanodine Receptor, While a Dominant Opposing Ca2+/Ba2+ Current Is Present
Source: Int J Mol Sci. 2024 Dec 29;26(1):203. doi: 10.3390/ijms26010203 (PMC11719481; doi:10.3390/ijms26010203)
Supplement: Supplementary file 1 [file ijms-26-00203-s001.zip › ijms-3326231-supplementary.pdf]

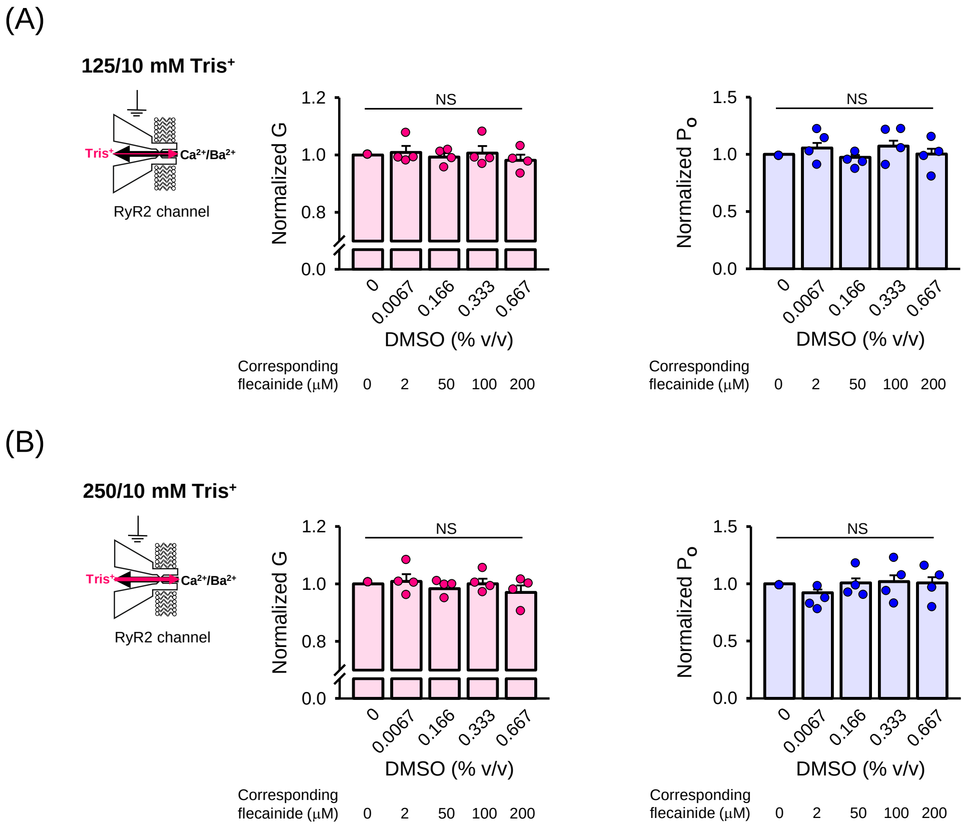

Figure S1. DMSO does not affect the RyR2-mediated countercurrent. DMSO of 0.0067, 0.166, 0.333, and 0.667% (v/v) was added to the cis compartment corresponding to the cytosol. These DMSO concentrations are equivalent to those present when flecainide of 2, 50, 100, and 200 μM was tested, respectively. When 125/10 mM (**A**) or 250/10 mM $Tris^+$ gradient (**B**) was utilized to drive the $Tris^+$ countercurrent in the cytosol-to-SR lumen direction, while a dominant Ca^{2+}/Ba^{2+} current in the opposite direction flowed through the RyR2 channel, no significant changes in G (**A**,**B**, left) and Po were observed (**A**,**B**, right). Data are shown as average ± SEM from n = 4 independent experiments. NS indicates not significant.

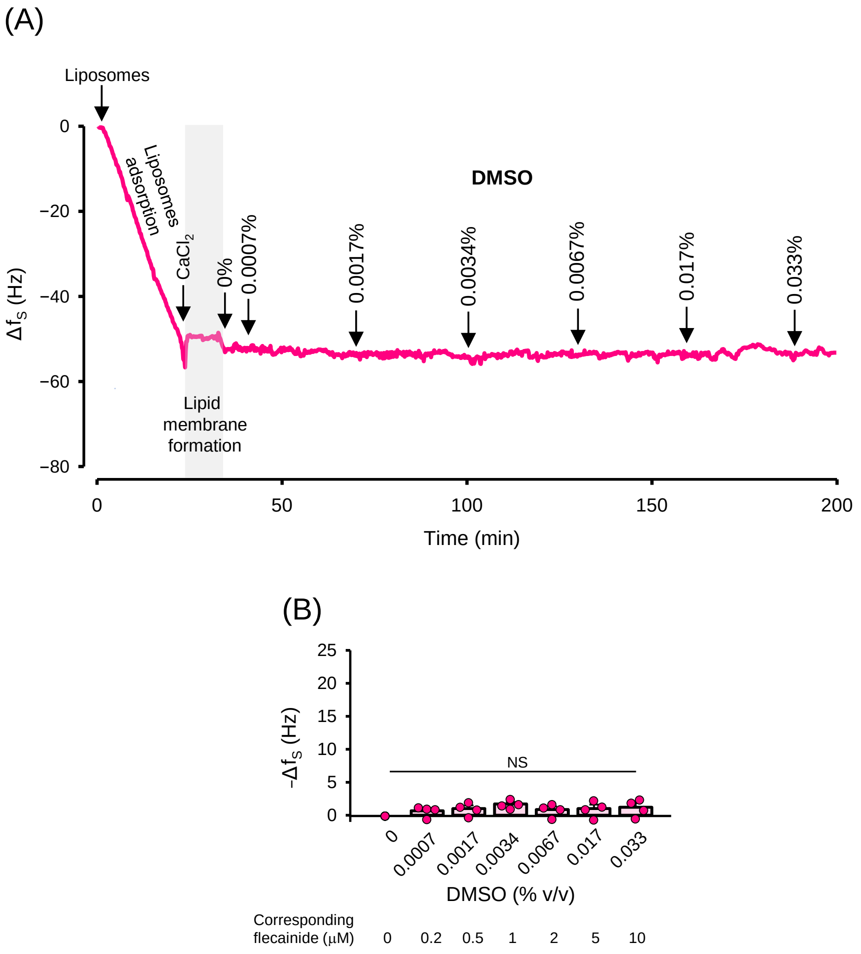

Figure S2. DMSO does not interact with the lipid membrane supported on a SiO_2 surface of the QCM sensor. (**A**) Representative response signal of the SiO_2 QCM sensor when the lipid membrane was formed over the sensor surface, followed by injection of 0.0007, 0.0017, 0.0034, 0.0067, 0.017, and 0.033% (v/v) DMSO. These concentrations are equivalent to those present when flecainide of 0.2, 0.5, 1, 2, 5, and 10 μM was tested, respectively. Downward arrows indicate times of DMSO additions. (**B**) The DMSO-dependence of $-\Delta f_S$. The data indicate a negligible interaction between DMSO and the lipid membrane. Data are shown as average ± SEM from n = 4 independent experiments. NS indicates not significant.
